# Evaluating 20-session augmented depression therapy for complex depression (ADepT+) versus usual care: a study protocol for a feasibility randomised controlled trial

**DOI:** 10.1136/bmjopen-2026-127811

**Published:** 2026-10-21

**Authors:** Laura Warbrick, Katie Marchant, Paul Moran, Kim A Wright, Sarah Morgan-Trimmer, Gordon Taylor, Rachel Hayes, Nigel Reed, John L Campbell, Barney D Dunn

**Affiliations:** 1 Mood Disorders Centre, University of Exeter Faculty of Health and Life Sciences, Exeter, UK; 2 Bristol Medical School, University of Bristol, Bristol, UK; 3 University of Southampton School of Primary Care Population Science and Medical Education, Southampton, UK; 4 College of Medicine and Health, University of Exeter, Exeter, UK; 5 Exeter Collaboration for Academic Primary Care, University of Exeter Faculty of Health and Life Sciences, Exeter, UK

**Keywords:** Depression & mood disorders, Psychosocial Intervention, Personality disorders, Randomized Controlled Trial, MENTAL HEALTH

## Abstract

**Introduction:**

Current depression psychotherapies are not optimised for individuals with co-occurring identity, emotional and interpersonal difficulties (sometimes conceptualised as personality difficulties or disorder), which we term ‘complex depression’. There is a need to develop enhanced psychotherapies for complex depression that repair symptoms, functioning and well-being. A novel wellbeing-focused therapy was codesigned with experts by experience: Augmented Depression Therapy for complex depression (ADepT+).

**Methods and analysis:**

This mixed-methods feasibility trial aims to recruit 60 participants with complex depression, randomised 1:1 to receive ADepT+ (20 weekly acute and five booster sessions) and usual care or usual care alone. Assessments will be conducted at intake, 8, 14 and 20 months post-randomisation. Candidate primary outcomes for a future trial include Patient Health Questionnaire-9 depression severity, SWEMWBS well-being and recovery of quality of life-10 quality of life. The candidate primary endpoint is 8 months post randomisation (when acute treatment should be completed).

Feasibility and acceptability of the trial procedures will be assessed against predetermined progression criteria regarding recruitment, retention, safety, intervention acceptability and intervention engagement. To evaluate proof-of-concept, within-arm and between-arm treatment effect sizes on outcomes at each assessment point will be reported. Novel Bayesian analyses will explore the potential inferiority, equivalence and superiority of ADepT+ compared with usual care. A process evaluation will explore implementation, potential mechanisms of impact and contextual modifiers of ADepT+.

**Ethics and dissemination:**

This study has been reviewed and approved by the UK Health Research Authority Research Ethics Service and South Leicester NHS Ethics Committee (IRAS: 337878). Publication within a peer-reviewed open access journal will be sought to disseminate study findings. Findings will be used to establish whether a future, larger trial is feasible and to refine the evaluation procedures and intervention.

**Registration:**

This study was prospectively registered on the ISRCTN registry (https://www.isrctn.com/ISRCTN62819149), prior to the start of recruitment. This paper describes the trial protocol (V.1.3) and was submitted before the final participant was recruited.

**Trial registration number:**

ISRCTN62819149.

**STRENGTHS AND LIMITATIONS OF THIS STUDY**

- This feasibility randomised controlled trial will provide information about the feasibility of conducting a definitive randomised controlled trial evaluating Augmented Depression Therapy for complex depression.
- Patient and public involvement is embedded in all aspects of the study, including codesign of the intervention.
- The single-site design is a limitation to the study methodology. The mixed-methods design will enable trial and treatment acceptability to be explored both quantitatively and qualitatively, improving potential generalisability of findings.
- Since there is no established treatment pathway for people with complex depression, an active control condition is not used.

## Background

 Depression has the highest incidence of all mental health problems, representing a significant cause of worldwide disability, disease burden and individual suffering.^1–3^ While talking therapies are effective for many people with depression, greater than 50% of individuals do not respond to evidence-based psychotherapy, such as cognitive behavioural therapy (CBT).^4^ Of those that do recover, >50% subsequently experience relapse or recurrence.^5^ Given the heterogeneous nature of depression, one possible way to improve care is to develop targeted treatments for discrete, clinically meaningful presentations marked by prognostically important characteristics associated with unequal care access.^6 7^

One presentation that may benefit from an enhanced depression intervention is those experiencing depression marked by additional difficulties regulating emotions, relationships and their identity (eg, 8–10). These difficulties likely arise in part due to exposure to childhood trauma and/or poverty exposure.^11 12^ While the personality disorder construct remains controversial (eg, 12 13) these difficulties are often conceptualised as mild or sub-threshold symptoms on the personality disorder continuum,^14 15^ resulting in significant stigma and unequal care access.^16–19^ We will henceforth refer to this presentation as ‘complex depression’. This complexity is an underlying vulnerability for more severe, chronic or recurrent depression^20–22^ which is characterised by elevated anhedonia,^23^ suicidality,^24^ well-being and functional deficits.^25^ However, distinction is needed between complex depression and those presenting with risk and symptom severity consistent with moderate-severe personality disorder^14^; for whom specialist treatment focused on these difficulties in multi-disciplinary settings is recommended.^26^

Those with complex depression are more likely to not respond or be denied access to depression treatment. Currently, in the UK, complex depression is typically assessed and treated within primary care (NHS talking therapies; NHS-TT).^27^ However, many individuals with complex depression are deemed too complex and discharged after assessment. For those who are offered therapy in this setting, personality difficulties predict poorer response to treatment.^28–30^ In both instances, there is typically no further treatment since secondary mental health resources are largely reserved for those with severe and enduring mental illness.^17^

There is an opportunity to enhance depression care by developing tailored treatments for complex depression. Such interventions should focus on repairing anhedonia, well-being and functioning. They should target key complex depression features that may maintain depression: emotion regulation and interpersonal skills deficits; difficulties managing relationships including the therapeutic alliance; and maladaptive ways of relating to self and others. Finally, they should accommodate broader characteristics of complex depression and thus be: trauma-informed; equipped to manage elevated suicidality; of sufficient intensity for more severe depression; and suitable for those with chronic, relapsing and/or difficult-to-treat depression.

Augmented Depression Therapy (ADepT)^31 32^ is a promising candidate intervention for complex depression, since it was co-designed to target anhedonia, well-being and functional recovery alongside depressive symptom relief. ADepT is structured into high-intensity weekly sessions suitable for more severe depression followed by flexible booster sessions aiming to reduce relapse/recurrence. ADepT involves identifying and moving towards values-consistent goals which may support people with complex depression to helpfully clarify their self-identity. This is augmented by the integration of evidence-based techniques targeting key individual maintaining factors including emotion dysregulation and interpersonal difficulties. The positive, solution-focused therapeutic style aims to build and sustain a positive therapeutic relationship and foster positive change in self-identity and responses to new challenges. Emerging findings indicate ADepT shows promising outcomes for depression with elevated anhedonia. For example, a pilot randomised controlled trial compared ADepT to CBT for anhedonic depression (n=82), found ADepT feasible and acceptable and with promising signal of potential clinical- and cost-effectiveness relative to CBT.^32^ Despite potential suitability and promising clinical outcomes, ADepT was not developed with complex depression in mind.

A co-design process with experts-by-experience and therapists was undertaken to adapt ADepT for complex depression (see ^33^). In particular, this considered the management of trauma exposure and elevated suicidality. A modified intervention for complex depression (ADepT+) resulted from this process. ADepT+ consists of an extended weekly treatment course (20 acute+5 booster sessions); affording more time and flexibility to deliver the intervention. This includes a longer assessment to acknowledge the role of past experiences/trauma in current difficulties and for proactive safety planning. A preliminary evaluation of ADepT+ in a multiple baseline case series of twenty-eight people with complex depression met all pre-specified progression criteria examining acceptability, feasibility, safety and efficacy.^33^

Further evaluation of whether ADepT+ is clinically and cost-effective for complex depression is justified. However, some key uncertainties about the trial design need to first be resolved by conducting a feasibility study before progressing to a definitive trial.^34^

### Objectives

The overarching aim of this study is to evaluate the safety, feasibility and acceptability of conducting a future definitive randomised trial determining the clinical and cost-effectiveness of ADepT+.

Specifically, the study will:

1. Assess the feasibility of recruitment (whether n=60 people with complex depression are willing to be recruited to a trial and randomised to Usual Care (UC) and ADepT+ or UC alone).
2. Assess the feasibility of participant retention.
3. Assess the safety of trial procedures (including the intervention).
4. Assess acceptability, feasibility and engagement with the intervention.
5. Characterise usual care provision overall and by group.
6. Inform future trial design by assessing overall acceptability and feasibility of evaluation procedures.

Progression to a definitive trial will be assessed against progression criteria informed by the Avery and colleagues’ guidance,^35^ presented in table 1. Further assessment of the overall acceptability and feasibility of evaluation procedures (including the intervention) is presented in online supplemental file 1.

**Table 1:** Criteria for progression to a definitive trial

		Red	Amber	Green
1	Proportion of target sample recruited	<50% of the target sample size is recruited	50–69% of the target sample size is recruited	≥70% of the target sample size is recruited
2	Candidate primary outcome data availability at primary endpoint (8 months)	<50% availability	50–69% data availability, with clear learning for improvement	≥70% data availability
3	Safety of intervention and trial procedures as agreed by TSC and sponsor	≥1 SAE potentially related to trial or treatment, with TSC agree procedures cannot be made safe	≥1 SAE potentially related to trial or treatment, but TSC agree procedures can be made safe	No trial or treatment related SAEs and TSC agree safe
4	Proportion of intervention arm rating therapy as acceptable (defined as rating intervention as acceptable, relevant and they would recommend to others at 8 months)	<50% indicate therapy is acceptable	50–69% indicate therapy is acceptable	≥70% indicate therapy is acceptable
5	Proportion of intervention group receiving minimum adequate dose defined as 50% of acute sessions (≥10 sessions) of therapy	<50% receiving a minimum adequate dose	50–69% receiving a minimum adequate dose, with clear learning for improvement	≥70% receiving a minimum adequate dose

Red: Do not progress to the main trial (unless TSC agree exceptional circumstances and a mitigation plan is present; amber: progress if action plan to mitigate problems can be determined and agreed with the TSC; green: progress directly to the main trial.

SAE, serious adverse events; TSC, Trial Steering Committee.

In addition to the primary feasibility objectives, this study aims to assess proof-of-concept that ADepT+ shows potential to be clinically effective; and explore the intervention logic.

## Method

This feasibility study protocol is reported according to the SPIRIT 2025 statement (online supplemental file 2).

### Design

This is a feasibility study, with a two-arm, randomised, parallel group, controlled trial design. Participants will be randomised on a 1:1 ratio to UC alone (control arm) or Usual Care and the novel intervention (ADepT+; intervention arm). Outcome measures will be recorded at baseline and at three follow-up points: 8, 14 and 20 months post randomisation. The primary endpoint is 8 months post-randomisation, intended to align with the expected end of the acute intervention. The study is of mixed-methods design and will integrate qualitative and quantitative findings exploring trial and treatment acceptability as well as the intervention logic.

### Setting and participants

The trial will take place in a single UK site (a specialist NHS outpatient psychological therapy clinic based at the University of Exeter) for details of the study site see: https://www.isrctn.com/ISRCTN62819149.

Eligible participants will be aged 18 or over with a diagnosis of Major Depression, according to DSM-5^36^ criteria assessed using the Structured Clinical Interview for DSM-5 (SCID-5) ^37^ and by scoring ≥10 on the Patient Health Questionnaire-9 (PHQ-9^38^: see online supplemental file 2 for details of outcome measures). Participants will also report personality (emotional, interpersonal, identity or impulsivity regulation) difficulties/disorder symptoms, assessed by scoring ≥9 on the Personality Disorder Severity ICD-11 (PDS-ICD-11)^14 39^ scale. Participants must be willing and able to give informed consent for participation and be registered with a general practice within the study site catchment area that they are willing to be informed of their participation. Participants must have sufficient written and spoken English to be able to make use of therapy and to be able to complete all research procedures and treatment without the need of a translator and to have completed the intake measures.

Participants will not be included if they are currently receiving psychosocial therapy; inpatient mental healthcare; or are unable to regularly attend therapy sessions within the site area. Participants will be excluded if they have a current/historic diagnosis of Bipolar-I disorder; lifetime experience of psychosis, assessed using the Mini International Neuropsychiatric Interview^40^; current substance dependence according to the International Classification of Diseases, 11th revision (ICD-11)^41^; pose an acute risk to themself or others; have a current moderate-severe personality disorder, or another co-occurring difficulty where these: (i) cannot be safely managed in a primary care outpatient setting; (ii) would significantly interfere with engagement in therapy; or (iii) are currently indicated to be the most appropriate primary focus of intervention .

Use of psychotropic medication will be recorded by self-report but will not inform eligibility.

### Sample size

Since this study is a feasibility trial, a sample size calculation was not performed. A total of 60 individuals will be recruited, since this was considered to evaluate the feasibility of trial procedures, including recruitment, uptake and attrition. A sample of 60 will allow estimation of loss to follow-up within ±12 percentage points, assuming 20% attrition.^42^

### Randomisation, concealment and blinding

Randomisation will be on a 1:1 ratio to intervention or control, by a pre-generated static list embedded within the trial database. The list will be generated by a statistician using randomised permuted blocks to ensure balance between arms. To ensure concealment, the randomisation sequence will be embedded within the database by a member of the Exeter Clinical Trials Unit independent of the management of the trial. Only unblinded researchers will have access to the database randomisation module and permissions to randomise a participant once eligibility is confirmed. The randomisation sequence will not be accessible by any member of the research team involved in enrolment. Participants will be informed of their allocation by an unblinded researcher; and be offered a post-randomisation follow-up call with an unblinded researcher within approximately 1 week of being informed of their allocation.

Blinding of participants to treatment allocation is not possible. Follow-up assessments will be conducted by researchers who are blind to the participant’s allocation. At follow-up, researchers will be instructed to maintain blindness by reminding participants not to disclose their allocation during the contact. Any unwitting participant disclosures will be recorded. After each follow-up assessment, the researcher will record which treatment they believe the participant received to evaluate success of the concealment procedures. Blindness will be maintained as far as possible; and deliberate unblinding will occur only in exceptional circumstances when the knowledge of the treatment arm is deemed essential to the researchers’ management of the patient (eg, certain Serious Adverse Events). Any unblinding will be recorded within the database and where possible a member of the research team who remains blinded to participant allocation will conduct future research assessment contacts with that participant. Since most outcomes are self-reported, this will apply only to a minority of clinician-rated follow-up outcomes.

### Recruitment

Recruitment aims to run for 12 months with a target recruitment rate of 5 participants per month; expecting some variation at the start of recruitment and during seasonal holiday periods.

Potential participants will be identified via self-referral to the study, by clinician signposting/referral to the study, and via electronic record search. Local NHS services, including but not limited to NHS-TT services, general practices and secondary care mental health teams will be informed of the study and how clinicians can direct potential participants to the study. Advertisements for the study will be placed in healthcare settings, public spaces and through traditional and social media and third sector organisations. Potential participants will be able to contact the research team directly, via telephone, email or post; or by completing a ‘permission to contact’ form which their clinician can share with the research team on their behalf. Initial format and outcome of enquiries and their outcome to inform the recruitment strategy of a future definitive trial.

After initial contact or referral, researchers will answer any questions and have an initial discussion with the potential participant if they wish. Following receipt of the completed ‘permission to contact’ form, a screening call will be arranged. In this call, the researcher will answer any questions and discuss details of the study; before going through the inclusion and exclusion criteria to determine whether it is likely the participant will be eligible. Those likely to be eligible and wishing to continue will be invited to the intake assessment; where written consent will be taken if the participant wishes to proceed, before conducting the eligibility assessment. Only if the participant is eligible to participate, is fully informed and has consented will they be enrolled in the study.

Participants will be offered an honorarium of £20 for participation and reimbursed for relevant travel expenses for each of the four assessments and the qualitative interview if completed. Participants allocated to the ADepT+ arm will not be reimbursed for attending treatment sessions.

### Intervention

ADepT+^33^ is a well-being and recovery-oriented psychotherapy for complex depression, modified from the emerging ADepT intervention for anhedonic depression^31 32^ using a co-design process (see ^33^). The treatment consists of a 20-session weekly acute treatment phase for clients with complex depression. This is followed by up to five optional booster sessions over the subsequent 12 months. Each session lasts approximately 60 min, structured by: agenda setting, feedback, homework setting and consolidation of learning. Sessions are structured into three phases, each focusing on different technical elements which are presented in table 2. ADepT+ involves identifying values and set goals consistent with these. While pursuing these goals, clients are supported to identify cognitive, behavioural and interpersonal mechanisms (eg, rumination, avoidance, interpersonal dampening; respectively) which maintain their depression and inhibit well-being gains. These are targeted by personalised delivery of ‘change techniques’ to promote adaptive mechanisms which promote ‘resilience’ (responses to challenges that minimise negative impacts) and ‘thriving’ (responses to opportunities that maximise benefits). A key component of ADepT+ is the interpersonal style implemented by therapists, which attends explicitly to the positive, and is solution- and future-focused. In contrast to CBT, where therapists primarily attend to and formulate the problem; ADepT+ therapists will focus on and formulate strengths and positive exceptions. This style aims to build and sustain a positive therapeutic relationship; model resilience, thriving and adaptive ways of relating to the self and others through judicious therapeutic self-disclosure.

**Table 2:** An overview of technical elements and adaptations for complex depression in each therapy phase of ADepT+

Therapy phase	Technical elements	Key adaptations for complex depression
Phase one (*approx. 8 sessions*)	Case conceptualisation. Safety planning. Introducing the ADepT rationale. Supporting clients to explore/clarify their values across domains (relationships, vocation, hobbies and self-care). Setting values-consistent goals. Behaviourally activating clients towards these in manageable steps.	(Universal) proactively safety planning in initial assessment sessions, using solution-focused principles, including completing novel safety planning therapy tool. Reviewing or updating this during therapy as needed and integrating this into well-being plan in late sessions. Integrating links between past experiences (including trauma) with current difficulties during case conceptualisation. More explicitly validating difficulties in current and past life. Framing values work as ‘exploration’ rather than ‘clarification’; and revisiting and revising values and goal setting elements in middle and late sessions. Introducing therapist style using a more graded exposure approach.
Phase two ‘Change’ sessions (*approx. 10 sessions*)	Identifying psychopathological processes which may be inhibiting well-being (pleasure, meaning, connection) benefits or maintaining depression while approaching these goals. Flexible delivery of cognitive, behavioural, interpersonal and emotional change techniques to promote adaptive mechanisms which increase ‘thriving’ (maximising benefits of activities) and ‘resilience’ (minimising negative impacts of challenges).	(universal) Considering current context/environment when mapping challenges or opportunities, or planning behavioural experiments; including using modified ADepT+ ‘mapping tool’ which includes a box to record considerations. Being explicitly validating of the impact or constraints of current context on difficulties and / or behavioural experiments within therapist style.
Phase three (*approx. 2 sessions +5 booster sessions*)	Consolidating learning. Planning for future well-being (to sustain and build on positive changes and recovery beyond therapy).	Tapering final acute sessions before transition to booster phase. Offering booster sessions on an ad-hoc basis to respond to significant setbacks or mood dips.

* Unless specified as universal, modifications are intended to be implemented according to individual need, based on the complex depression presentation.

ADepT+, augmented depression therapy for complex depression.

The key adaptations for complex depression iADepT+ are outlined within table 2. ADepT+ includes safety planning component, which is completed with all clients during initial sessions, to ensure a proactive, solution-focused risk management plan is in place should this escalate during therapy. This plan is revisited and revised as needed during treatment and is ultimately integrated into the comprehensive well-being plan created in later sessions. ADepT+ is primarily intended to be delivered in a personalised, flexible fashion according to the individual complex depression presentation. For example, for clients with complex depression who have difficulties with an overly fixed identity, or an unstable sense of self, therapists should frame values work in early sessions as ‘exploring’ rather than ‘clarifying’. In middle and/or later sessions, therapists should return to revise and clarify values, supporting clients to integrate new learning about their values, identity and how they relate to others gained during therapy. Detailed therapist guidance notes describing the implementation of these key modifications, and other minor adaptations in ADepT+ for complex depression are presented in AlSayed Nasser and colleagues^33^ online supplemental materials.

A provisional logic model of the ADepT+ intervention is presented in online supplemental file 3.

#### ADepT+ therapists

At least three therapists will deliver the intervention. They will all be qualified in cognitive behavioural, behavioural or dialectical behavioural therapy and have prior experience delivering ADepT and of working with people with mood disorders. Therapists receive weekly, 90-min supervision within a small group format, facilitated by an ADepT supervisor.

#### Engagement, fidelity and competence

All sessions will be audio-recorded (where participants consent to this) to allow for assessment of therapist competence and fidelity to the ADepT+ protocol and to support supervision. Four tapes from each therapist (each from a different client) will be randomly selected for competency and fidelity assessment by an ADepT+ trial therapist or supervisor (with no therapists rating their own tapes). The ADepT competency rating tool, developed within the previous pilot trial of ADepT (see ^32 43^), will be used to rate the tapes. Competency is defined as scoring three or more on each ADepT domain: style, structure and technique from 0 (‘incompetent’) to 6 (‘expert’); and an overall competency rating is achieved by averaging these scores. Fidelity is rated separately on a scale from 0 to 9. All selected tapes will be rated by two assessors to determine inter-rater reliability. ADepT+ session attendance and mode of attendance will be recorded within clinical notes. Therapists will attempt to contact and reengage participants if they do not attend a session without explanation (DNA). In line with local policy, participants will be discharged from ongoing care if they DNA three consecutive sessions and will be considered an unscheduled discontinuation.

### Usual care

Those in the active condition will receive the intervention in addition to usual care (ADepT+). In line with recommended best practice, those allocated to ADepT+ will be advised not to engage in another psychosocial therapy during the acute weekly treatment phase of the intervention. There will be no restrictions placed on the usual care for participants allocated to ADepT+ within the booster phase, nor in the event of early discontinuation of the intervention.

Those in the control condition will receive UC alone; since there is no established care pathway or therapy recommendation for people with complex depression. No restrictions will be placed on participants allocated to the control condition, including accessing other psychosocial therapies once enrolled in the study.

### Outcomes and measures

The primary outcomes of this study relate to the overarching feasibility objectives and assessment against the pre-specified progression criteria (see table 1). The candidate primary end point will be 8 months post-randomisation (when all participants are anticipated to have completed acute therapy).

The secondary candidate endpoints will be 14 months post-randomisation and 20 months post-randomisation (when all intervention participants are anticipated to have completed the booster therapy phase). Figure 1 presents the measures collected at each time point. A detailed description of each quantitative measure is presented in online supplemental file 4.

**Figure 1:**
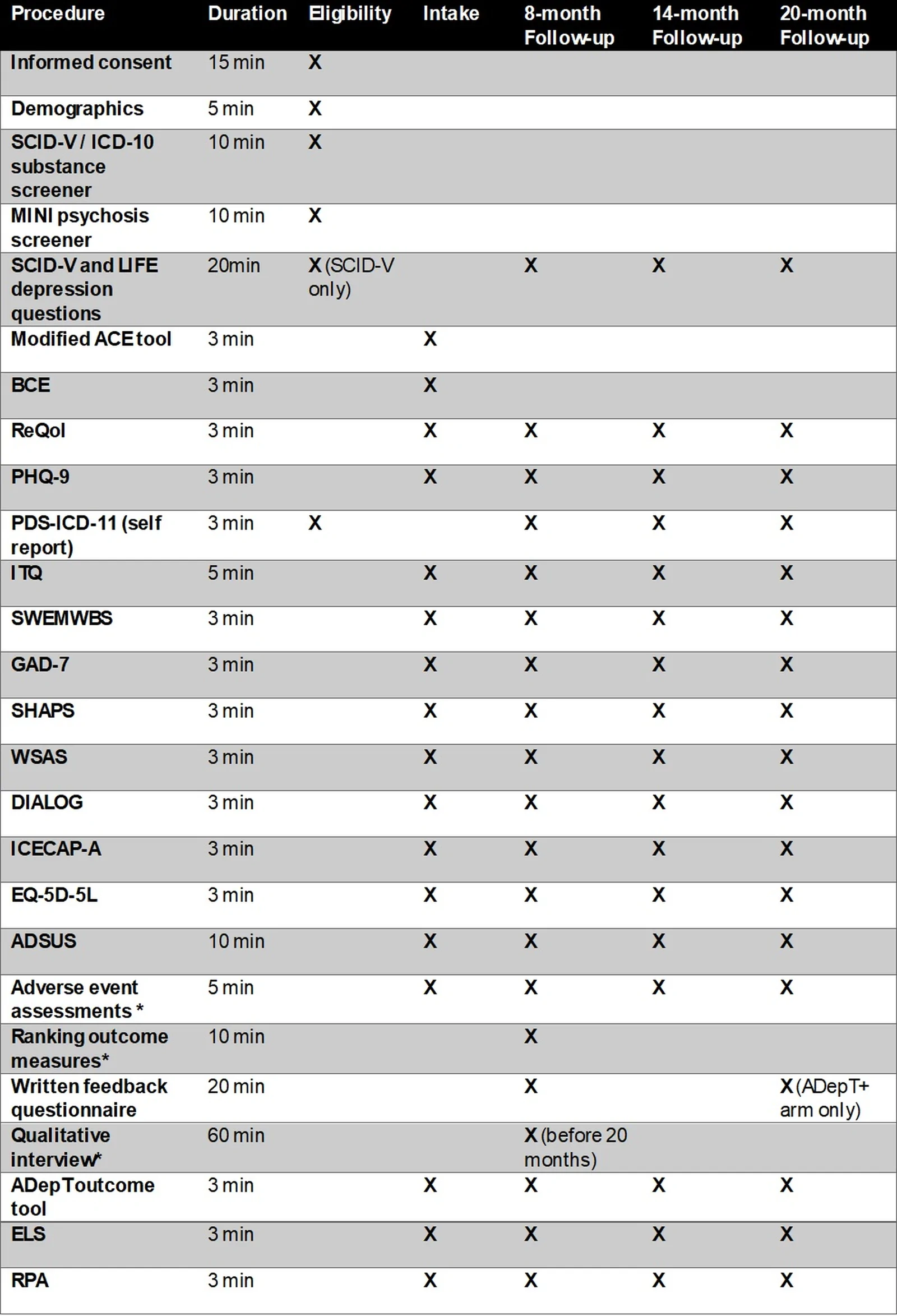
Schedule of procedures, showing outcomes collected at each timepoint. ACE; adverse childhood experience, ADepT; augmented depression therapy, ADSUS; adult service use schedule, BCE; Benevolent childhood experiences, ‘DIALOG’; scale measuring life and treatment satisfaction, ELD; engaged living scale, EQ-5D-5L; EuroQol five dimensions questionnaire, GAD-7; General Anxiety Disorder 7-item scale, ICD-10; International disease classification 10th edition, ICECAP-A; ICEpop capability measure for adults, ITQ; international trauma questionnaire, LIFE; longitudinal interval follow-up evaluation, MINI; mini international neuropsychiatric interview, PDS-ICD-11; personality disorder severity ICD-11, PHQ-9; patient health questionnaire 9, ReQoL; recovery of quality of life, RPA; responses to positive affect, SCID-V; structured clinical interview for DSM-V, SHAPS; Snaith Hamilton pleasure scale, WSAS; work and social adjustment scale.

#### Demographic information

At the intake eligibility assessment, demographic information will be collected, capturing: age, self-reported gender identity and sex assigned at birth, ethnicity, relationship status, highest level of education, perceived household financial status, postcode deprivation indices, if currently open to secondary care services, previous episodes of therapy (date, duration and type of therapy, and whether or not they found it helpful), current psychiatric medication use (name, dose, duration and concordance). Positive and adverse early life experiences will be captured using the Benevolent childhood experiences questionnaire^44^ and modified short Adverse Childhood Experience tool.^45^

#### Clinical outcomes

The following self-reported clinical outcome measures will be completed at baseline, 8, 14 and 20 months post-randomisation (in order of presentation): depression, captured by PHQ-9^38^; personality difficulty/disorder symptoms, captured by the PDS-ICD-11^14 39^; recovery of quality of life, as measured by the recovery of quality of life-10 (ReQoL-10^46^); Post-Traumatic Stress Disorder (PTSD) and complex PTSD symptoms, indexed by the International Trauma Questionnaire^47^; well-being measured by SWEMWBS^48^; anxiety, as indexed by the Generalized Anxiety Disorder 7-item scale (GAD-7^49^), anhedonia as measured by the Snaith Hamilton Pleasure Scale,^50^, the Work and Social Adjustment Scale^51^ indexing functional impairment, and ‘DIALOG’, a scale measuring life and treatment satisfaction.^52^

At baseline, depression status will also be captured by the clinician-administered SCID-5 Major Depression module.^37^ At all subsequent follow-ups, this module will be accompanied by the clinician-administered Longitudinal Interval Follow-up Evaluation,^53^ capturing DSM-V depression diagnostic status over the follow-up interval.

To make it possible to assess symptom change over time during the acute treatment phase, participants in the ADepT+ arm only will additionally complete the PHQ-9, GAD-7 and SWEWMBS before each session.

The candidate primary clinical outcomes for a subsequent trial are the PHQ-9, ReQoL-10 and SWEMWBS.

#### Health economic outcomes

Health and service care use will be captured using the Adult Service Use Schedule (see ^54 55^) to capture health and social care service use. Health related quality of life will be captured using the EuroQol five dimensions questionnaire,^56^ and by the ICEpop Capability measure for Adults.^57^

#### Quantitative process measures

Candidate process measures will be completed by all participants at intake and 8, 14 and 20 months post-randomisation, as well as following session 4, 8, 12, 16 and 20 in the intervention group. These will measure self-reported values-based living and behaviours using the Engaged Living Scale^58^ and the bespoke ADepT Outcome Scale; as well as capturing how participants appraise positive moods with the Responses to Positive Affect scale.^59^ In the ADepT+ group only, these will also be captured following session 4, 8, 12, 16 and 20 and include the Working Alliance Inventory- Short Revised Form^60^ to measure the therapeutic alliance.

#### Additional measures

The following measures will be collected using bespoke measures (copies available at: https://www.isrctn.com/ISRCTN62819149):

At 8 months, in an Outcome Measure Ranking questionnaire all participants will rank candidate primary and secondary clinical outcome measures according to which are most relevant to their difficulties, and they would most wish to experience changes in.

An ‘asking about adverse events’ form will be completed by researchers after the intake assessment, each follow-up and after any contact with participants where there is any indication of a potential adverse event.

An 8-item quantitative and qualitative questionnaire will capture experiences of seeking and receiving usual care at each follow-up. In addition, those in the control arm who have received active treatment in usual care will rate acceptability, relevance, and whether they would recommend to others the usual care they received at each follow-up (on 5-point scale).

A qualitative questionnaire will capture acceptability and experiences of the research processes at 8 months. In addition, at 8 and 20 months, intervention arm participants will rate acceptability, relevance and likelihood they would recommend the ADepT+ intervention to others (each on a 5-point scale), as well as describe in brief written format qualitative experiences of weekly therapy (at 8 months) and booster sessions (at 20 months). At 8 months only, they will rate perceived changes in depression, anxiety, well-being, pleasure, positive mood and negative mood since starting ADepT+ (on a scale from 1 to 7) and how worthwhile therapy was considering these changes (on a scale from 1 to 5).

With participant consent, on early exit from the study participants will be invited to complete a brief qualitative questionnaire asking about their experience of the research.

Therapist participants will rate ADepT+ in terms of clinical effectiveness, enjoyment in practicing, suitability for and whether they would recommend NHS implementation (on a scale from 1 to 5). They will also rate whether they agree training built their knowledge, skill and confidence in key style, structure and technical element competencies (on a scale from 1 to 5).

#### Qualitative interviews

Between 8 and 20 months post-randomisation, a subset of up to 15 participants from the intervention group will be invited to a qualitative interview, purposively sampled to span diversity in demographic characteristics, intake clinical characteristics, clinical benefit, therapy and acceptability ratings of the therapy. All trial therapists will be invited to interview at the end of their delivery of therapy within the study.

Interviews will be conducted face-to-face or remotely (videoconferencing or via telephone) according to participant preference, be semi-structured, audio recorded and take approximately 60 min. Interviews will be conducted by an unblinded member of the study team who has received training in qualitative interviewing.

In advance of the interview, participants will be given the option to create a visual timeline of their experience of the therapy. Where participants opted to complete this, interviews will begin by discussing the visual timeline, and/or by a topic guide informed by the intervention logic model (see online supplemental file 2). Interviews will explore the implementation of the intervention; potential mechanisms of impact; and how contextual factors affect how the intervention is implemented, and impact on participants.

### Procedure

Informed consent will be taken prior to, or at the start of the eligibility assessment by a member of the research team; and will be verbally reaffirmed prior to qualitative interviews. Following completion of the eligibility assessment and baseline measures, participants will be randomised to either Usual Care alone or with the addition of ADepT+. The intervention group will begin treatment as soon as possible after randomisation; allowing for local administrative processes and coordination. We anticipate the first appointment to take place approximately 2 weeks after randomisation; aiming for this to take no longer than 4 weeks. Assessments at 8, 14 and 20 months will involve completion of self-report measures online, as well as completing clinician-administered measures with a trained, blinded researcher in person or remotely (telephone or videoconferencing) according to their preference. Self-report measures and questionnaires will be collected and managed online, using Research Electronic Data Capture (REDCap).^61^ Where this is not possible, or due to patient preference, data will be captured by pen and paper CRF and then entered on REDCap by the research team. Clinician-rated follow-up measures will be completed via phone as default. Assessment format and questionnaire completion format will be tailored according to individual preferences, as discussed at the intake assessment.

### Adverse events

All adverse events will be assessed for seriousness, causality and expectedness by the Principal Investigator and will be recorded and reported from the point of randomisation until the 20 month follow-up assessment or the point of withdrawal from the study. Hospitalisations for elective treatment of a pre-existing condition will not be reported as serious adverse events. Symptoms of Depression/Personality Disorder/Complex depression, including suicidal ideation and self-harm themselves will not be defined as adverse events, however deterioration in mental state resulting in emergency support access; and self-harm or suicidal behaviour requiring medical attention will be classed as an AE.

Given the increased contact with the study team in the intervention group, we may expect higher rates of AEs reported relative to the control group. Therefore, we will place particular attention on AEs that are judged to be related to therapy or study participation.

### Data analysis

Data will be analysed on an intention to treat (according to their randomised group irrespective of treatment actually received) and complete case basis, except where otherwise stated. Analyses will include observed data only; no imputation of missing outcome data will be performed. As this is a feasibility trial with a small sample size, effect sizes (and their 95% CIs) rather than statistical significance will be reported in all analyses. Mean (SD) values for all continuous variables and number (%) values for all categorical variables will be reported at each assessment for the overall sample and as a function of arm.

Primary data analysis will focus on a descriptive analysis of the feasibility objectives of the study. We will report the number (% of target) recruited into the trial, and length and mean monthly recruitment rate. We will report retention and clinical, health economic and process measure completion rates at each timepoint, overall and by group. Sample characteristics at intake will be reported descriptively for each arm.

Outcome measures and calculated binary metrics (reliable improvement, deterioration, remission and reliably and clinically significant change) will be reported descriptively for each arm at intake and at each follow-up (eg, 8–14 and 20 month), alongside raw change from intake. Within-arm paired samples t-tests will estimate effect size change for each outcome at each timepoint by arm (relative to intake). Quantitative intervention acceptability and attendance data will be reported descriptively.

Serious adverse events (SAEs) will be reported for the 20-month trial period using the ‘as treated’ approach: meaning participants in the ADepT+ arm will be classified as ‘usual care’ if they do not attend at least one ADepT+ therapy session. Reporting will distinguish between SAEs potentially related to trial procedures (excluding the intervention) vs those related to the intervention. SAEs will be reported numerically, and as the number and percentage of individual participants experiencing one or more SAE during the trial period. To further assess potential harms, rates of reliable deterioration in each clinical outcome measure will be calculated overall and as a function of arm for each follow-up, relative to intake.

Intervention and usual care costs will be estimated and reported descriptively by arm alongside calculated quality-adjusted life years for each timepoint.

Exploratory analyses investigating clinical proof-of-concept will follow those conducted in a previous pilot trial of the original ADepT intervention for anhedonic depression.^32^ Within-arm continuous effects and calculated binary metrics will be compared with those from a previous evaluation of ADepT+, the original ADepT pilot trial and within-arm effects observed for similar interventions on similar populations in the extant trials literature. Between group differences at 8 months in continuous outcomes will be estimated using linear regressions (controlling for intake levels of the dependent variable), reporting raw score (m, SD) and standardised effect size (Cohen’s d, 95% CI) differences. For categorical outcomes, binary logistic regressions will estimate the odds ratio (OR; 95% CI) differences between arms at 8 months. In each case, CIs will be inspected to assess potential signal of inferiority or superiority of ADepT+ relative to UC, (using minimum clinically important difference (MCID) threshold for continuous outcomes).

For candidate primary outcomes at 8 months, we will run exploratory Bayesian analyses to plot the probability of difference between arms, and interpret the probability of inferiority, equivalence and superiority of ADepT+ relative to UC at the MCID threshold.

A detailed statistical analysis plan will be published on the trial registration (https://www.isrctn.com/ISRCTN62819149) prior to preparing the data for analysis.

### Qualitative data analysis

Qualitative analyses will evaluate the feasibility and acceptability of the evaluation procedures and explore the intervention logic. Qualitative interviews will be audio-recorded and then transcribed by a member of the study team or with a confidentiality agreement in place by a suitable individual working with the team.

To explore the feasibility and acceptability of the evaluation procedures, written qualitative responses to feedback questionnaires will be closely read and coded using a framework approach, informed by the feasibility objectives and Sekhon and colleagues’ framework for assessing intervention acceptability.^62 63^ Joint display will be used to integrate reporting of quantitative and qualitative acceptability findings.

To explore the intervention logic, qualitative interview data will be analysed using a thematic analysis approach,^64^ focused on exploring how the intervention was implemented, potential mechanisms of impact and contextual. A detailed analysis plan of the process evaluation methodology including integration of quantitative and qualitative data on the trial registration prior to analysis.

### Patient and public involvement

People with personal experience, or have a relative with depression and complex depression have co-designed the original intervention and the guidance for adapted delivery for complex depression; and have been involved in all aspects of the study design and protocol including the: funding application; trial design; choice, quantity and intervals of outcome measure collection; inclusion and exclusion criteria; materials for participants such as the information sheet, consent form and interview topic guide; as well as the recruitment strategy; and will be consulted about any substantial modifications or modifications of important detail during study delivery, and all aspects of dissemination. Lived experience co-author KM has contributed to all the preliminary work and initial design of the study and will be a member of the research team and involved in all aspects of the study delivery throughout the project lifespan, including all aspects of dissemination. An independent public contributor will have membership of the Trial Steering Committee (TSC).

### Trial governance

The TSC has been convened, including members independent of the sponsor and funder, which will also perform the functions of a Data Monitoring Committee (reviewing data collected including adverse events and making recommendations for the future conduct of the study), given the small scale of the study. The TSC is chaired by Dr Peter Taylor (University of Manchester) and is attended by the CI (LW). See trial registry for full details of TSC membership.

The TSC will be responsible for advising the study team on the conduct of the study. The TSC will periodically review overall safety data to determine patterns and trends of events, or to identify safety issues, which would not be apparent on an individual case basis. All safety relevant events will be reported to the TSC and reviewed on an ongoing basis. The TSC will meet approximately twice per year over the life of the study.

Sponsor and relevant national regulatory approval will be for any substantive changes to the protocol, which will be recorded publicly via update to the ISRCTN registration record.

## Ethics and dissemination

The conduct of the trial will be in accordance with the Helsinki Declaration. The study has received approval from the UK Health Research Authority Research Ethics Service (IRAS ID: 337878), and from all relevant local approval bodies.

Prior to consent, participants will receive full information about the study, the opportunity to consider the information, and to discuss the study at a screening call, before and during the intake assessment with a researcher. Researchers will be trained in taking informed consent, including capacity assessment where appropriate. Only individuals with capacity to make an informed decision on participation will be able to consent to take part.

The data arising from the study will be the property of the University of Exeter and the CI. On completion, the data will be analysed and a Final Study Report prepared. The full study report will be made available in open access form via the University of Exeter. Findings will be disseminated via seeking publication within a peer-reviewed open access journal and conference presentations; as well as written summaries and public engagement events; aiming to reach local, national and international audiences; including members of the public, clinicians, healthcare decision makers and academics. Participants will be asked to state whether they wish to receive a copy of the findings which they will be sent once these have been finalised. To protect participant anonymity in this small number study, the raw data will not be made publicly accessible.

The full study protocol, data collection forms and intervention manual and materials can be requested from the corresponding author.

Data sharing is not applicable to this study protocol, since no datasets have been generated or analysed.

## Discussion

This feasibility study is a randomised controlled trial aiming to determine the feasibility of conducting a large randomised controlled trial determining the clinical and cost-effectiveness of ADepT+. The study is of mixed-methods design where acceptability of the evaluation procedures is evaluated through both quantitative measures of attendance, completion and acceptability ratings, as well as from qualitative interview and questionnaire data. The embedded mixed-methods process evaluation aims to explore for who, in what context the intervention may lead to change; to refine the therapy protocol theory of change which underpins it.

The study builds on co-design of the intervention to adapt it for complex depression, and a case series where 28 people with complex depression received the intervention. The case series met all pre-specified progression criteria, enabling continuation to the current study. Performance against the progression criteria described here will determine continuation to a future definitive trial. Should a future trial establish effectiveness, the intention is this could be implemented within routine care in the UK to address the unmet mental health needs of people with complex depression.

### Limitations

The study will take place in a single site, meaning feasibility findings may not generalise to additional sites in a future definitive trial. The mixed-methods design aims to bring contextual depth through integration of qualitative data, improving potential generalisability.

Inherent within the feasibility design, this study was not designed, and does not have sufficient statistical power to assess clinical effectiveness of the intervention. Instead, intervention proof-of-concept will be examined within exploratory analyses investigating potential clinical signal.

## Supplementary material

10.1136/bmjopen-2026-127811 online supplemental file 1
