# Supplementary material for: Evaluating 20-session augmented depression therapy for complex depression (ADepT+) versus usual care: a study protocol for a feasibility randomised controlled trial
Source: BMJ Open. 2026 Oct 21;16(10):e127811. doi: 10.1136/bmjopen-2026-127811 (PMC13691877; doi:10.1136/bmjopen-2026-127811)
Supplement: Supplementary file 1 [file bmjopen-2026-127811.reviewer_comments.pdf]

PEER REVIEW HISTORY

BMJ Open publishes all reviews undertaken for accepted manuscripts. Reviewers are asked to complete a checklist review form and are provided with free text boxes to elaborate on their assessment. These free text comments are reproduced below.

ARTICLE DETAILS

Title (Provisional)

Evaluating 20-session Augmented Depression Therapy for Complex Depression (ADepT+) versus usual care: a study protocol for a feasibility randomised controlled trial

Authors

Warbrick, Laura; Marchant, Katie; Moran, Paul; Wright, Kim A; Morgan-Trimmer, Sarah; Taylor, Gordon; Hayes, Rachel; Reed, Nigel; Campbell, John L; Dunn, Barney D

This article was not externally reviewed at BMJ Open. Protocols that have been independently assessed prior to submission to BMJ Open are usually fast-tracked to publication on the grounds that further substantial changes will not be possible. This independent assessment will usually be external, independent review for both a substantial grant award from a non-commercial or government funder and ethics approval.
