# Supplementary material for: Evaluating 20-session augmented depression therapy for complex depression (ADepT+) versus usual care: a study protocol for a feasibility randomised controlled trial
Source: BMJ Open. 2026 Oct 21;16(10):e127811. doi: 10.1136/bmjopen-2026-127811 (PMC13691877; doi:10.1136/bmjopen-2026-127811)
Supplement: Supplementary file 2 [file bmjopen-16-10-s001.docx]

# Supplementary File 1. *Feasibility Objectives and their means of (quantitative) measurement*

|  | ***Feasibility objectives*** | ***Means of (quantitative) Measurement*** |
| --- | --- | --- |
| **1** | **Recruitment** |  |
| Assess feasibility of recruitment (whether n=60 people with complex depression are willing to be recruited to a trial and randomised to Usual Care (UC) and ADepT+ or UC alone). | | To assess *progression criteria one*, we will report the number (% of target sample size) recruited into the trial.  To further assess recruitment, we will report the overall length and mean (SD) monthly rate of recruitment. |
| 1.1 | Inform future trial design by assessing overall acceptability and feasibility of recruitment procedures (including randomisation). (Including adequacy to recruit a sample consistent with complex depression presentation; and sufficient diversity and inclusivity in sample) | The CONSORT diagram will be interrogated to assess the number of individuals, identified, approached, consented and randomised (calculating conversion % from each step of the pipeline to the next and reporting reasons for exclusion at each stage of the pipeline descriptively). A recruitment plot will display the cumulative number of participants recruited each month against the corresponding cumulative recruitment target.  To examine effectiveness of different recruitment methods, the number (%) of individuals identified, approached, consented, completed baseline assessment, and randomised for each separate recruitment (e.g., self-referral, clinician referral, clinician signposting, electronic record search) method will be tabulated. Challenges to recruitment identified by the research team during the trial will be narratively summarized. To characterize the nature of the sample recruited, intake demographic and clinical characteristics of the sample will be reported and examined, including establishing the severity of depression and personality difficulty/disorder reported, assessing what proportion of the sample would meet ‘difficult-to-treat depression’ criteria, and evaluating the extent to which a diverse and inclusive sample has been achieved.  To assess acceptability of the randomisation procedure, we will report the number (%) of individuals in each arm withdrawing at each stage of the trial who specifically stated it was because of concerns about the randomisation process or the arm they were allocated to. This data, along with rates of any cause early exit from the trial in each arm, will be inspected for any evidence of imbalance between arms in attrition rate.  The mean (SD) answers to acceptability, satisfaction, and recommend ratings of participants in the UC arm, along with the number (%) of participants in the UC arm who agreed care was acceptable, relevant, and would recommend it to others, will be reported (i.e., answering ‘very’ or ‘extremely’ acceptable and relevant, and ‘likely’ or ‘very likely’ to recommend to others; pooled across these categories). |
| **2** | **Retention** |  |
| Assess the feasibility of participant retention | | To assess *progression criteria two*, the number (%) of participants randomised with complete data at both intake and 8-month (primary-end point) assessment on all candidate primary outcome measures will be reported (overall and as a function of arm).  To further assess retention procedures, the consort diagram will be interrogated to assess the number (%) of individuals retained overall and in each arm at each follow-up, with reasons for exclusion or drop-out reported descriptively. In addition, we will report the number (%) of participants with complete data on each individual outcome measure (all clinical, process evaluation, and health economic variables) at each assessment point. |
| **3** | **Safety** |  |
| Assess the safety of the trial procedures (including the intervention). | | To assess *progression criteria three*, the pooled number of potentially trial- and treatment-related SAEs will be calculated. The TSC safety report and conclusions will be narratively summarised for each SAE, including TSC judgement of relatedness to study procedures (including intervention) and if related, whether procedures could be made safe in a future trial.  To further examine trial and intervention safety, AEs and SAEs will be summarised descriptively (overall and by group); including: relatedness to trial procedures (excluding the intervention), relatedness to ADepT+ intervention, seriousness, severity and expectedness. We will report (separately) the number of trial-related, treatment-treatment related and unrelated AEs and SAEs, and the number (%) of participants experiencing one or more trial-related, treatment-related and unrelated AE and SAE over the 20-month trial period for the overall sample and as a function of arm.  Rates of reliable deterioration on each clinical outcome measure at each follow-up assessment (relative to baseline) will be computed, overall and as a function of arm. We will report the number (%) showing reliable deterioration on one or more outcome measure at each time point and at one or more timepoint overall and as a function of arm.  We will also seek administrator, clinician and supervisor feedback on the internal risk management policies used to respond to disclosures of self-harm, suicidality or broader risk to self or others during the trial. |
| **4** | **Intervention** |  |
| Assess acceptability, feasibility and engagement with the intervention | | |
| 4.1 | Establish overall therapy acceptability. (*progression criteria four*) | To assess *progression criteria four*, the number (%) of participants in the ADepT+ arm who rate ADepT+ as acceptable, relevant, and that they would recommend ADepT to others will be reported (i.e., answering ‘very’ or ‘extremely’ relevant and acceptable, and ‘likely’ or ‘very likely’ to recommend to others; pooled across these categories). We will also report the number (%) of each response type for each question separately. |
| 4.2 | Assess engagement with ADepT+. (*progression criteria five*) | To assess *progression criteria five*, the number (%) of participants in the ADepT+ arm who attend a minimum adequate dose (at least 10 acute treatment sessions, equating to ≥50% of the intended acute treatment dose) will be reported.  To further assess ADepT+ intervention engagement, we will report the mode and mean (SD) number of acute and booster sessions marked attended, client cancelled, therapist cancelled, and DNA. We will classify the number (%) of participants: completing a full acute dose of ADepT with a planned discharge, choosing to curtail acute treatment but having a planned discharge, dropping out of acute treatment (unscheduled discontinuation); and those no longer suitable for the treatment setting who were referred to another service (without completing acute treatment); and attending at least one booster session. |
| 4.3 | Assess feasibility of candidate endpoints. | To assess the suitability of eight-month primary end point and twenty-month secondary endpoint, we will report the mean and median length of time (in weeks) to complete the acute ADepT+ phase (randomisation to final acute session) and the booster phase (final acute session to final booster session). In addition, we will report the cumulative number (%) of participants completing acute treatment phase as a function of weeks since randomisation and completing booster treatment as a function of weeks since acute treatment end. |
| 4.4 | Assess therapist fidelity and competence and associated data collection procedures (to inform refinement of the therapist training and supervision). | To evaluate and refine how well procedures and tools rating ADepT+ sessions are functioning, we will assess the number (%) of clients who give consent for sessions to be recorded and the number (%) of sessions where the recording is captured in a way that can then be coded.  We will report: the number (%) of therapists rated as competent (scoring at least 3 on style, structure, technique and overall ratings for all tapes rated); the number (%) of therapists rated as showing adequate fidelity to the protocol (scoring > 5 on fidelity ratings for all sessions); mean (SD) scores across therapists and tapes of style, structure, competence, overall, and fidelity ratings; and the number (%) of therapists classified at each competence level based on average overall rating across tapes on the competency scale (0-1 = incompetent; 1 -2 = novice; 2 -3 = advanced beginner; 3-4 = competent; 4 -5 = proficient, 5-6 = expert). We will also report the number (%) of clients who completed acute treatment and dropped of treatment of those allocated to each therapist. |
| **5** | **Usual care** |  |
| Characterise experiences of usual care provision overall and by group. | | A key uncertainty to resolve is the degree to which participants access other active mental health treatments (especially other psychotherapies) as part of usual care. ADSUS health care utilisation data and answers to a bespoke mental health treatment utilization questionnaire will be examined to determine the number (%) of participants in each arm engaging with other active mental health treatment during the initial eight-month follow-up (acute treatment phase) and subsequent follow-ups. We will report the number (%) of participants reporting to have accessed active mental health treatments by group at each follow-up. We will narratively summarise treatment types, delivery settings, experiences or details of treatment reported and perceived similarity with the care they sought for their mental health. Where participants do report engaging with other active mental health treatments during the trial, we will report the number (%) of participants accessing support within different service contexts and narratively describe the treatment setting and treatment type, overall experiences or details of treatment and perceived fit with the care they sought, grouped by trial arm. Where participants do not report receiving active mental health treatments, we will narratively summarise the settings and treatment types of any care sought (but not received), alongside reported reasons for not accessing active treatment in usual care. |
| **6** | **Other trial procedures** |  |
| Inform future trial design by assessing overall acceptability and feasibility of evaluation procedures*. (For recruitment, retention and intervention procedures see previous sections)* | | |
| 6.1 | Assess feasibility of blinding concealment procedures. | To assess the adequacy of allocation concealment procedures, incidents of unblinding at each assessment will be reported descriptively (including what led to unblinding). We will report the number (%) of participants unblinded overall and the bang blind index for each assessment point, overall and by arm. |
| 6.2 | Inform primary outcome measurement selection for future trial. | Candidate primary outcome measures include: PHQ-9 depression severity, SWEMWBS wellbeing, and ReQoL-10 quality of life. Completion rates n (%) will be presented at each timepoint; participant feedback on measures will be analysed qualitatively. Participant rankings of these three clinical outcomes and five additional potential secondary outcome domains (personality difficulty symptoms, anxiety symptoms, anhedonia symptoms, social functioning and life / treatment satisfaction), will provide information on their value to participants. We will report mean (SD) rank for each domain, where lower score equals higher rank, as well as number (%) of participants selecting each domain as first or second highest rank.  To examine sensitivity to the impact of the intervention, raw score and change clinical outcome measures will be reported descriptively (m, SD) by group for each assessment point. In addition, rates of reliable improvement, reliable deterioration, remission (scoring beneath clinical cut off on the scale), response (>50% improvement relative to intake scores), and reliable and clinically significant change will be computed, with number (%) data reported on each outcome at each relevant assessment point in each arm. We will report raw change from intake for each outcome at each timepoint. Within-arm paired samples t-tests will estimate effect size change (Cohens’ d, 95% CI) for each outcome at each timepoint by arm (relative to intake).  This information will be considered within a stakeholder consensus meeting to agree the optimal primary (or co-primary) outcome for a future trial. The final decision will be made by the CI. |
| 6.3 | Assess feasibility of collecting health care resource utilisation data health and wellbeing-related quality of life data. | Intervention and usual care costs will be estimated and reported descriptively by arm for each outcome. Patterns of missingness in resource use data will be explored, and resource use categories prone to missing data / being misunderstood for self-report measures will be reported descriptively. We will report the number (%) cases with sufficient data to calculate Quality-Adjusted Life Years (QALYs) at each timepoint overall and by group. We will calculate quality-adjusted life years for each and report descriptively by arm at each timepoint. Participant feedback on health economic data collection procedures. These will inform whether there is a need to develop strategies to collect missing data and/or to include proxy assessments in the main trial. |
| 6.4 | Assess the feasibility of quantitative process measure data collection procedures | Per measure completion rates at each timepoint. Participant feedback on process measure data collection procedures. |

**Supplementary File 2: *SPIRIT 2025 checklist of items to address in a randomized trial protocol****

| **Section / Topic** | **No** | **SPIRIT 2025 checklist item description** | **Reported on page no.** |
| --- | --- | --- | --- |
| **Administrative information** | | |  |
| Title and structured summary | 1a | Title stating the trial design, population, and interventions, with identification as a protocol | 1 |
|  | 1b | Structured summary of trial design and methods, including items from the World Health Organization Trial Registration Data Set | 2 |
| Protocol version | 2 | Version date and identifier | 2 |
| Roles and responsibilities | 3a | Names, affiliations, and roles of protocol contributors | 1 |
|  | 3b | Name and contact information for the trial sponsor | 15 |
|  | 3c | Role of trial sponsor and funders in design, conduct, analysis, and reporting of trial; including any authority over these activities | 14 |
|  | 3d | Composition, roles, and responsibilities of the coordinating site, steering committee, endpoint adjudication committee, data management team, and other individuals or groups overseeing the trial, if applicable | 1, 13-14; ISRCTN trial registry |
| **Open science** | | |  |
| Trial registration | 4 | Name of trial registry, identifying number (with URL), and date of registration. If not yet registered, name of intended registry | 2 |
| Protocol and statistical analysis plan | 5 | Where the trial protocol and statistical analysis plan can be accessed | 12, 14 |
| Data sharing | 6 | Where and how the individual de-identified participant data (including data dictionary), statistical code, and any other materials will be accessible | 14 |
| Funding and conflicts of interest | 7a | Sources of funding and other support (e.g., supply of drugs) | 15 |
|  | 7b | Financial and other conflicts of interest for principal investigators and steering committee members | 15 |
| Dissemination policy | 8 | Plans to communicate trial results to participants, healthcare professionals, the public, and other relevant groups (e.g., reporting in trial registry, plain language summary, publication) | 14 |
| **Introduction** | | |  |
| Background and rationale | 9a | Scientific background and rationale, including summary of relevant studies (published and unpublished) examining benefits and harms for each intervention | 1-2 |
|  | 9b | Explanation for choice of comparator | 8 |
| Objectives | 10 | Specific objectives related to benefits and harms | 4 |
| **Methods: Patient and public involvement, trial design** | | |  |
| Patient and public involvement | 11 | Details of, or plans for, patient or public involvement in the design, conduct, and reporting of the trial | 13 |
| Trial design | 12 | Description of trial design including type of trial (e.g., parallel group, crossover), allocation ratio, and framework (e.g., superiority, equivalence, non-inferiority, exploratory) | 5 |
| **Methods: Participants, interventions, and outcomes** | | |  |
| Trial setting | 13 | Settings (e.g., community, hospital) and locations (e.g., countries, sites) where the trial will be conducted | 5 |
| Eligibility criteria | 14a | Eligibility criteria for participants | 5 |
|  | 14b | If applicable, eligibility criteria for sites and for individuals who will deliver the interventions (e.g., surgeons, physiotherapists) | 7 |
| Intervention and comparator | 15a | Intervention and comparator with sufficient details to allow replication including how, when, and by whom they will be administered. If relevant, where additional materials describing the intervention and comparator (e.g., intervention manual) can be accessed | 7-8 |
|  | 15b | Criteria for discontinuing or modifying allocated intervention/comparator for a trial participant (e.g., drug dose change in response to harms, participant request, or improving/worsening disease) | Supplementary File 5, Section 3-4 |
|  | 15c | Strategies to improve adherence to intervention/comparator protocols, if applicable, and any procedures for monitoring adherence (e.g., drug tablet return, sessions attended) | 8 |
|  | 15d | Concomitant care that is permitted or prohibited during the trial | 8 |
| Outcomes | 16 | Primary and secondary outcomes, including the specific measurement variable (e.g., systolic blood pressure), analysis metric (e.g., change from baseline, final value, time to event), method of aggregation (e.g., median, proportion), and time point for each outcome | 8-11 |
| Harms | 17 | How harms are defined and will be assessed (e.g., systematically, non-systematically) | 11, 12, Supplementary File 1, Supplementary File 5, Section 2 |
| Participant timeline | 18 | Time schedule of enrollment, interventions (including any run-ins and washouts), assessments, and visits for participants. A schematic diagram is highly recommended (see Figure) | 10-11, Figure 1. |
| Sample size | 19 | How sample size was determined, including all assumptions supporting the sample size calculation | 5 |
| Recruitment | 20 | Strategies for achieving adequate participant enrollment to reach target sample size | 6 |
| **Methods: Assignment of interventions** | | |  |
| Randomization: |  |  |  |
| Sequence generation | 21a | Who will generate the random allocation sequence and the method used | 6 |
|  | 21b | Type of randomization (simple or restricted) and details of any factors for stratification. To reduce predictability of a random sequence, other details of any planned restriction (e.g., blocking) should be provided in a separate document that is unavailable to those who enroll participants or assign interventions | 6 |
| Allocation concealment  mechanism | 22 | Mechanism used to implement the random allocation sequence (e.g., central computer/telephone; sequentially numbered, opaque, sealed containers), describing any steps to conceal the sequence until interventions are assigned | 6 |
| Implementation | 23 | Whether the personnel who will enroll and those who will assign participants to the interventions will have access to the random allocation sequence | 6 |
| Blinding | 24a | Who will be blinded after assignment to interventions (e.g., participants, care providers, outcome assessors, data analysts) | 6 |
|  | 24b | If blinded, how blinding will be achieved and description of the similarity of interventions | 6 |
|  | 24c | If blinded, circumstances under which unblinding is permissible, and procedure for revealing a participant’s allocated intervention during the trial | 6 |
| **Methods: Data collection, management, and analysis** | | |  |
| Data collection methods | 25a | Plans for assessment and collection of trial data, including any related processes to promote data quality (e.g., duplicate measurements, training of assessors) and a description of trial instruments (e.g., questionnaires, laboratory tests) along with their reliability and validity, if known. Reference to where data collection forms can be accessed, if not in the protocol | 10, Supplementary File 2 |
|  | 25b | Plans to promote participant retention and complete follow-up, including list of any outcome data to be collected for participants who discontinue or deviate from intervention protocols | 10, 11 |
| Data management | 26 | Plans for data entry, coding, security, and storage, including any related processes to promote data quality (e.g., double data entry; range checks for data values). Reference to where details of data management procedures can be accessed, if not in the protocol | Supplementary File 5, Section 1 |
| Statistical methods | 27a | Statistical methods used to compare groups for primary and secondary outcomes, including harms | 11-12, Supplementary File 1. |
|  | 27b | Definition of who will be included in each analysis (e.g., all randomized participants), and in which group | 11 |
|  | 27c | How missing data will be handled in the analysis | 11 |
|  | 27d | Methods for any additional analyses (e.g., subgroup and sensitivity analyses) | 12 |
| **Methods: Monitoring** | | |  |
| Data monitoring committee | 28a | Composition of data monitoring committee (DMC); summary of its role and reporting structure; statement of whether it is independent from the sponsor and funder; conflicts of interest and reference to where further details about its charter can be found, if not in the protocol. Alternatively, an explanation of why a DMC is not needed | 13, ISRCTN trial registry |
|  | 28b | Explanation of any interim analyses and stopping guidelines, including who will have access to these interim results and make the final decision to terminate the trial | Supplementary File 5, Section 2 |
| Trial monitoring | 29 | Frequency and procedures for monitoring trial conduct. If there is no monitoring, give explanation | 13 |
| **Ethics** | | |  |
| Research ethics approval | 30 | Plans for seeking research ethics committee/institutional review board approval | 13 |
| Protocol amendments | 31 | Plans for communicating important protocol modifications to relevant parties | 13 |
| Consent or assent | 32a | Who will obtain informed consent or assent from potential trial participants or authorized proxies, and how | 14 |
|  | 32b | Additional consent provisions for collection and use of participant data and biological specimens in ancillary studies, if applicable | NA |
| Confidentiality | 33 | How personal information about potential and enrolled participants will be collected, shared, and maintained in order to protect confidentiality before, during, and after the trial | Supplementary File 5, Section 1 |
| Ancillary and post-trial care | 34 | Provisions, if any, for ancillary and post-trial care, and for compensation to those who suffer harm from trial participation | Supplementary File 5, Section 2 |

References:

Chan A-W, Boutron I, Hopewell S, Moher D, Schulz KF, et al. SPIRIT 2025 statement: updated guideline for protocols of randomised trials. BMJ 2025;389:e081477. <https://dx.doi.org/10.1136/bmj-2024-081477>

# Supplementary File 3. Provisional logic model of ADepT+ for complex depression

**Supplementary File 4 *Details of quantitative intake and outcome measures used.***

*Title, construct addressed and scale and item details quantitative measures.*

| Measure Title (Acronym) | Construct Addressed | Scale and Item Details |
| --- | --- | --- |
| Benevolent childhood experiences questionnaire (BCE) | Positive developmental experiences before age 18 | Self-report. 10 items rated ‘No’, ‘Yes’ or ‘Prefer not to answer’ |
| Modified Center for Disease Control and Prevention short Adverse Childhood Experience (ACE) tool | Number and frequency of adverse experiences in childhood (items 1-12). Frequency of benevolent experiences (items 13-15). Modified to include an additional item 16 to report if there were any other adverse experiences not otherwise covered. | Self-report. First 6 items rated ‘No’, ‘Yes’.  Items 7-12 rated ‘Never’, ‘Once’ or ‘More than Once’.  Items 13-15 rated on a 5-point scale from ‘Never’ to ‘All of the time’.  Modified additional item 16 rated ‘No’ or Yes’.  All items include option to answer ‘Don’t know/not sure’ or ‘Prefer not to answer’. |
| Recovering Quality of Life tool (ReQoL-10) | Recovery of quality of life for people with mental health difficulties | Self report. 10-items rated on a 5-point scale from 0 (None of the time) to 4 (Most or all of the time). |
| Patient Health Questionnaire 9 (PHQ-9) | Current depression symptoms | Self-report. 9-items rated on a 4-point scale from 0 (Not at all) to 3 (Nearly every day) |
| Personality Disorder Severity ICD-11 scale (PDS-ICD-11) | Current personality difficulty / disorder symptom severity | Self-report. 14 items. Items 1-10 are scored “2-1-0-1-2” to indicate the severity and direction of difficulties, and items 11-14 are rated on a 4-point scale from 0 (describing no difficulties in the item)-3 (describing the highest severity of difficulty in the item). |
| short Warwick-Edinburgh Mental Wellbeing Scale (SWEMWBS) | Wellbeing | Self-report. 7-items rated on a 5-point scale from 1 (None of the time) to 5 (All of the time). |
| International Trauma Questionnaire (ITQ) | Current Post Traumatic Stress Disorder (PTSD) and Complex Post-traumatic Stress Disorder (CPTSD) symptoms. | Self-report. 18-items rated on a 5-point scale from 0 (Not at all) to 4 (Extremely) |
| Generalised Anxiety Disorder Scale (GAD-7) | Current anxiety symptoms | Self report. 7-items rated from on a 4-point scale from 0 (Not at all) to 3 (nearly every day) |
| Snaith Hamilton Pleasure Scale (SHAPS) | Current Anhedonia symptoms | Self-report. 14-items rated on a 4-point scale from 1 (Definitely Agree) to 4 (Definitely Disagree) |
| Work and Social Adjustment Scale (WSAS) | Functioning in key life domains | Self-report. 5-items rated on a 9-point scale from 0 (Not at all) to 8 (Very severely). |
| ‘DIALOG’ Scale | Satisfaction in key life domains for people with mental health difficulties | Self-report. 11-items rated on a 7-point scale from 1 (Totally dissatisfied) to 7 (Totally satisfied) |
| EuroQol five dimensions questionnaire (E5-5D-5L) | Health-related quality of life, for the calculation of Quality Adjusted Life Years (QALYs) | Self-report. 5 health domain items rated on 5-point scales, ranging from ‘no problems’; to complete loss of functioning or ‘extreme’ problems/symptoms. Plus rating of overall health today from 0 (worst health you can imagine) to 100 (best health you can imagine). |
| the ICEpop Capability measure for Adults (ICECAP-A) | Wellbeing-related quality of life, for the calculation of QALYs | Self-report. 5 wellbeing domain items rated on 4-point scales; ranging from experiencing the wellbeing domain ‘a lot’ or in ‘all areas of life’; to being unable to experience the wellbeing domain in any area of life. |
| Responses to Positive Affect scale (RPA [71] | Typical responses to positive affect (e.g. dampening and amplifying appraisals) | 17-item self-report questionnaire rated on a 4-point scale from 1 (Almost never) to 4 (Almost always). |
| Engaged Living Scale (ELS[70]) | Values-based living and life fulfilment | 16-item self-report scale rated from 1 (Completely Disagree) to 5 (Completely Agree) |
| ADepT Outcome Tool (bespoke) | Capturing values clarification and aligned behaviour and appraisals relevant to the therapy logic model | 11-item self-report scale rated from 1 (Strongly disagree) to 5 (Strongly agree) |
| Working Alliance Inventory-Short Revised | Capturing patient-perceived quality of the therapeutic alliance | 12-item self-report scale rated from 1 (Seldom) to 5 (Always) |

**Supplementary File 5**

## Section 1: Data Management and storage

Data will be managed in accordance with General Data Protection Regulation [71]. Electronic study data will be stored on a secure sponsor-managed database platform, purpose built for the management of clinical research data.

Participants will be allocated a trial number at the point of consenting. Only those who require access to identifiable data (e.g. for the purposes of contacting the participant during their involvement in the study) will have access to this information. For all other purposes, the participant will be identified by their unique trial number.

By default, all data will be entered directly into the database by participants or researchers via eCRF. in circumstances where there is difficulty accessing the internet or necessary IT equipment, paper source data worksheets may be required with subsequent data entry by the local research team; or due to participant preference (for self-report measures). In this case, this data will be transcribed into the secure, online trial database by blinded trial personnel. Clinical records will be stored according to standard practice within the NHS service hosting the intervention. Hard copies of information / measures gathered as part of this research study will be anonymised and stored in a locked filing cabinet in a locked office in the Department of Psychology, University of Exeter or a locked cabinet in a locked office on Devon Partnership Trust premises (Research and Development Team base). Hard copy consent forms will be stored in a locked cabinet separately to study data. Audio recordings of qualitative interviews and therapy sessions will be recorded directly to, or immediately transferred in digital form to, a secure data storage area hosted by the University of Exeter (Secure Data Research Hub: SDRH). Personal data will be transferred and stored only where necessary.

Personally identifiable information (including qualitative interview audio recordings) will be stored for 12 months after the end of the study, allowing for data analysis and sharing of a results summary to participants who requested these at study entry. Audio recordings of therapy sessions will be stored for 12 months after the end of the study, allowing for data analysis and sharing of a results; except where additional consent was given for their use in future research and / or for training and teaching purposes, where they will be retained for a maximum of 20 years.

## Section 2: Adverse Events and Harms

Adverse events (AEs), as defined by the HRA as any untoward occurrence or worsening of an unintended sign, symptoms or disease that occurs during participation, regardless of any relation to participation; will be recorded. An AE will be classified as serious if it results in: death, life threatening injury or condition, permanent impairment to a body structure, body function or the capacity of the individual to function in daily life; hospitalisation or extension of existing hospitalisation; foetal death, or a congenital abnormality or birth defect.

Symptoms of Depression / Personality Disorder / Complex depression, including suicidal ideation and self-harm themselves are not defined as adverse events, however deterioration in mental state resulting in emergency support access; and self-harm or suicidal behaviour requiring medical attention will be classed as an AE.

The only expected reactions to participation are distress or symptom exacerbation (including behaviours such as self-harm, suicidal behaviour or substance use which may be considered part of an individual’s coping responses to distress / symptom exacerbation).

AE reports will be solicited at follow-up points and monitored during all research contacts, and self-report measure responses. Any AEs believed to be study related, or with potential to be classified as ‘severe’ or a SAE by researchers / therapists will be reported to the CI within 24h. Other AEs must be reported within 1 week. The reporting period is from consent until completion of the 20-month follow-up.

Given the increased contact with the study team in the intervention group, we may expect higher rates of AEs reported relative to the control group. Therefore, we will place particular attention on AEs that are judged to be related to therapy or study participation.

### Early Trial Discontinuation

Should an unexpected serious adverse reaction occur to either the therapy or the trial procedures, and this is judged to be directly related to trial participation or to the therapy, all non-clinical (therapy) procedures will be temporarily halted pending investigation and analysis of the extent to which future risk can be mitigated against. If it is judged that this is not possible, the trial will be discontinued. This process will be led by the sponsor in collaboration with the TSC chair and CI. In this event, onwards clinical care decisions for those in treatment will be decided by the clinical service on an individual basis.

Should information come to light from external sources that indicates that the therapy intervention or trial procedures are unsafe, the process outlined above will be followed.

### Indemnity

The sponsor, University of Exeter, will provide indemnity to meet the potential legal liability for harm to participants arising from the management, design or conduct of the research.

The sponsor has not made arrangements for payment of compensation in the event of harm to the research participants where no legal liability arises.

## Section 3: Withdrawal Criteria

Participants will be informed in the study information sheet that they may opt to withdraw from the study at any point, without giving a reason and without affecting their standard clinical care or access to future research. If participants withdraw from both the therapy and the trial, no further data will be collected from them (other than inviting them to give their views on the therapy via the feedback form or semi-structured interview). All data collected up until that point will be retained. If participants withdraw from the therapy, they will remain in the study, and continue to be invited to major research assessment points, unless they indicate that they also wish to withdraw from the study. Participants in the intervention arm can withdraw from the research without their access to care within the AccEPT Clinic being affected. In this event no further data will be collected from them (other than inviting them to give their views on the therapy via the feedback form or semi-structured interview); and any collection of therapy-related outcomes will take place within the clinical service.

Withdrawal criteria

1. Participant does not wish to continue with the study.
2. The participant experiences an event that is judged by the TSC to indicate discontinuation is in the best interests of the participant or sponsor.
3. Participant and / or research team believe that the intervention or trial participation will result in, or is likely to result in, a serious adverse reaction if continued.
4. Participants taking part in the semi-structured interviews about their experiences of the study will be given a 14 day period after the interview during which they can opt to withdraw their data if they wish, after this time, it is likely that the data analysis will have begun and it will not be possible to withdraw their data.
